# Blood Transfusion and Increased Perioperative Risk in Coronary Artery Bypass Grafts

**DOI:** 10.21470/1678-9741-2017-0034

**Published:** 2017

**Authors:** Igor C. Campos, Valessa Tanganelli, Hugo P. Maues, Marcio C. M. Coelho, Fernanda A. Martins, Giovana Munhoz, Julyana G. T. Egito, Hayala C. C. Souza, Cássio M. C. Giannini, Pedro S. Farsky

**Affiliations:** 1 Instituto Dante Pazzanese de Cardiologia, São Paulo, SP, Brazil.; 2 Laboratory of Statistics and Epidemiology, Instituto Dante Pazzanese de Cardiologia, São Paulo, SP, Brazil.; 3 Fundação Pró-Sangue, Instituto Dante Pazzanese de Cardiologia, São Paulo, SP, Brazil.; 4 Instituto Dante Pazzanese de Cardiologia; Universidade de Santo Amaro (UNISA), São Paulo, SP, Brazil.

**Keywords:** Blood transfusion, Bloodless Medical and Surgical Procedures, Myocardial Revascularization, Coronary Artery Bypass

## Abstract

**Objective:**

To correlate blood transfusions and clinical outcomes during hospitalization in coronary artery bypass grafting surgery (CABG).

**Methods:**

Transfusion, clinical and hematological data were collected for 1,378 patients undergoing isolated or combined CABG between January 2011 and December 2012. The effect of blood transfusions was evaluated through multivariate analysis to predict three co-primary outcomes: composite ischemic events, composite infectious complications and hospital mortality. Because higher risk patients receive more transfusions, the hospital mortality outcome was also tested on a stratum of low-risk patients to isolate the effect of preoperative risk on the results.

**Results:**

The transfusion rate was 63.9%. The use of blood products was associated with a higher incidence of the three coprimary outcomes: composite infectious complications (OR 2.67, 95% CI 1.70 to 4.19; *P*<0.001), composite ischemic events (OR 2.42, 95% CI 1.70 to 3.46; *P*<0.001) and hospital mortality (OR 3.07, 95% CI 1.53 to 6.13; *P*<0.001). When only patients with logistic EuroSCORE ≤ 2% were evaluated, *i.e.*, low-risk individuals, the mortality rate and the incidence of ischemic events and infectious complications composites remained higher among the transfused patients [6% *vs.* 0.4% (*P*<0.001), 11.7% *vs.* 24,3% (*P*<0.001) and 6.5% *vs.* 12.7% (*P*=0.002), respectively].

**Conclusion:**

The use of blood components in patients undergoing CABG was associated with ischemic events, infectious complications and hospital mortality, even in low-risk patients.

**Table:** 

Abbreviations, acronyms & symbols	
CABG	= Coronary artery bypass grafting surgery
CI	= Confidence interval
OR	= Odds ratio

## INTRODUCTION

The use of blood products in cardiac surgery is a known risk factor for adverse events during the perioperative period. Unnecessary transfusions can cause further increases in surgical risk and complications. Blood transfusion criteria in this scenario are not yet well established and exhibit variability between different centers and among professionals at the same center.

Several studies, mostly observational, have demonstrated an association between pre- and intraoperative anemia and increased morbidity and mortality after cardiac surgery^[1,2]^. A drop in hematocrit in the absence of shock has been the main indication for transfusion in critically ill patients, and in up to 29% of these cases there is no clear justification for transfusion^[3]^.

A potential indication of transfusion is the association between severe anemia and hypoxemia, which may increase postoperative risk, but this theory is not confirmed.

Patients receiving transfusions are usually at a greater preoperative risk, added with the risk of the event leading to transfusion. Preliminary studies show higher mortality rates, ischemic and infectious outcomes, however, these previous studies are not corrected for the individual risk of patients. Furthermore, these studies evaluated patients submitted to general surgery, and when evaluated patients undergoing cardiac surgery, are not specifically for coronary artery bypass surgery (CABG)^[4-6]^.

This study evaluates the association between blood transfusions and hospital morbidity and mortality in patients undergoing CABG (isolated or combined); furthermore, is analyzed a subgroup of low-risk patients, in order to minimize the effects of the greater preoperative risk of the transfused patients, identified by a low Logistic EuroSCORE.

## METHODS

Consecutive patients undergoing isolated or combined CABG between January 1^st^ 2011 and December 31^st^ 2012 were included in the study. Patients aged ≤ 18 years or those who were not undergoing surgery with a cardiopulmonary bypass were not selected.

The lowest hemoglobin values measured between a patient's departure from the operating room until 10 days after surgery were included. Intraoperative hemoglobin data were not included.

The transfusion of blood products (packed red cells, packed platelets, fresh frozen plasma and cryoprecipitate), conducted between 5 days before and 10 days after CABG, was considered to be of primary interest.

Blood transfusions were performed when hemoglobin levels fell below 7 g/dL or in critical clinical conditions, at the physician's discretion.

Three co-primary outcomes were pre-specified: infectious outcomes, ischemic outcomes, and hospital death.

An infectious outcome was defined as a composite of respiratory infection, wound infection and/or sepsis. An ischemic outcome was defined as a composite of stroke, transient ischemic attack, acute myocardial infarction and/or acute renal failure (an increase in the creatinine value of at least 50% as per the Acute Renal Injury Network^[7]^). Hospital mortality was defined as death that occurred within 30 days of surgery or during hospitalization^[8]^.

Demographic characteristics and comorbidities were obtained from institutional databases, and data were organized by absolute and relative frequencies (qualitative variables) and by mean and standard deviation (quantitative variables).

Information infrequently missing from the database (< 1%) was redistributed in the median or most frequent category. Information more frequently missing (> 1%) was considered void and was not included in analyses. The study was approved by the Ethics Committee of each institution involved.

### Statistical Analysis

To compare quantitative variables from transfused and non-transfused patients, Student's-t and Mann-Whitney tests were used according to the distribution of variables. To compare groups by categorical variables, Fisher's exact test was used.

Univariate analysis was performed between the transfusion and non-transfusion groups using preoperative variables. These variables were included, with data on the transfusion of blood products, in a multivariate logistic regression model to predict mortality, infectious outcomes and ischemic outcomes. The odds ratio (OR) and 95% confidence interval (CI) values were reported.

## RESULTS

A total of 1,389 patients underwent CABG during the study period. Eleven patients had no transfusion data and were excluded. Clinical characteristics are described in Table 1.

**Table 1:** Population characteristics (n=1,378).

Characteristic	Non-transfused (n=498)	Transfused (n=880)	*P* value
Age, n (%)			
< 65 years	336 (67.5)	441 (50.1)	< 0.001
65 to 75 years	136 (27.3)	308 (35)	__
≥ 75 years	26 (5.2)	131 (14.9)	__
Female, n (%)	77 (15.5)	354 (40.2)	< 0.001
Preoperative Hb, g/dL	14.4±1.5	13.6±1.7	< 0.001
Minimum Hb, g/dL	8.9±1.3	7.9±1.2	< 0.001
Weight, kg	80.6±14.1	72.8±14.1	< 0.001
Anoxia time, minutes	50.6±20.6	61.7±25.1	< 0.001
ECC time, minutes	74±29	93±38	< 0.001
Logistical EuroSCORE (%)	3.18±4.54	5.82±7.9	< 0.001
CrCl ≤ 30 mg/dL, n (%)	5 (1.1)	29 (3.5)	0.01
EF < 50%, n (%)	181 (36.8)	322 (37)	0.95
Diabetes mellitus, n (%)	199 (40)	371 (42.2)	0.45
SAH, n (%)	429 (86.1)	764 (86.8)	0.74
Smoker, n (%)			
Non-smoker	212 (42.6)	418 (47.5)	0.1
Current smoker	111 (22.3)	160 (18.2)	-
Ex-smoker (> 1 year)	175 (35.1)	302 (34.3)	-
COPD, n (%)	22 (4.4)	43 (4.9)	0.79
Prior stroke/TIA, n (%)	25 (5)	54 (6.1)	0.47
Carotid disease (> 50%), n (%)	20 (4)	54 (6.1)	0.1
Peripheral vascular disease, n (%)	38 (7.6)	82 (9.3)	0.32
AMI in last 30 days, n (%)	64 (12.9)	144 (16.4)	0.08
Combined MR, n (%)	54 (10.8)	188 (21.4)	< 0.001
Affected arteries, n (%)			
Uni or biarterial	146 (30.3)	205 (24.4)	0.02
Triarterial or trunk	336 (69.7)	635 (75.6)	-

AMI=acute myocardial infarction; COPD=chronic obstructive pulmonary disease; CrCl=creatinine clearance; ECC=extracorporeal circulation; EF=ejection fraction; Hb=hemoglobin; MR=myocardial revascularization surgery; SAH=systemic arterial hypertension; TIA=transitory ischemic attack

Among the 1,378 patients included, 498 (36.1%) did not receive any type of blood product transfusion and 880 (63.9%) received one or more. Of these, 63 (4.6% of the total) individuals received only blood components other than packed red blood cells, and 817 (59.3%) received at least one packed red blood cell product, with or without other blood products. The percentage of patients using each of the blood products studied and the mean numbers of transfused bags are listed in Table 2.

**Table 2:** Transfusion rates and mean transfused units.

	n (%)	Mean units ± SD
Packed red blood cells	817 (59.3)	2.37±1.68
Packed platelets	157 (11.4)	8.34±6.91
Cryoprecipitate	82 (6)	8.04±3.45
Fresh frozen plasma	274 (19.9)	2.77±2.12

SD=standard deviation

The transfused patients were older, had lower body weight, preoperative hemoglobin values and minimum hemoglobin values in the first 10 days after surgery, and had higher logistical EuroSCOREs than control patients. Anoxia and cardiopulmonary bypass times were significantly longer in this group. The proportion of women, number of patients with stage 4 or 5 kidney failure, and incidence of combined surgery and triarterial or trunk injuries were significantly higher among transfused patients.

The incidence of composite infectious outcomes among transfusion patients was 16.1% compared to 6% among the non-transfused (OR 3; 95% CI 1.99 to 4.53; *P*<0.001]. This association was also significant when each of its components was evaluated [sepsis 3% *vs.* 0.6% (OR 5.02, 95% CI 1.51 to 16.68; *P*<0.001), respiratory infection 8.6% *vs.* 2.6% (OR 3.53, 95% CI 1.94 to 6.42; *P*<0.001), surgical wound infection 9.7% *vs.* 3.6% (OR 2.77, 95% CI 1.59 to 4.80; *P* <0.001)].

Ischemic outcomes occurred in 29.4% of the transfused group and in 12.6% of the non-transfused (OR 2.91, 95% CI 2.13 to 3.98; *P*<0.001). As with infectious outcomes, there was also an association between transfusion and each component of ischemic outcomes [kidney failure 21% *vs.* 9.3% (OR 2.58, 95% CI 1.81 to 3.68; *P*<0.001), stroke 4.1% *vs.* 1.8% (OR 2.32, 95% CI 1.11 to 4.85; *P*<0.001), acute perioperative myocardial infarction 8% *vs.* 2.2% (OR 3.83, 95% CI 2.01 to 7.30; *P*<0.001)].

Hospital mortality among transfused patients was significantly higher than among non-transfused patients [10% *vs.* 2.2% (OR 4.92, 95% CI 2.6 to 9.3; *P*<0.001)].

In the low-risk group, patients identified by logistic EuroSCORE ≤ 2% (252 transfused and 260 non-transfused patients), had a mortality of 6% *vs.* 0.4% (*P*<0.001) for the transfused and non-transfused group, ischemic outcome of 11.7% *vs.* 24.3% (*P*<0.001) and infectious outcome 6.5% *vs.* 12.7% (*P*=0.002).

**Fig. 1:**
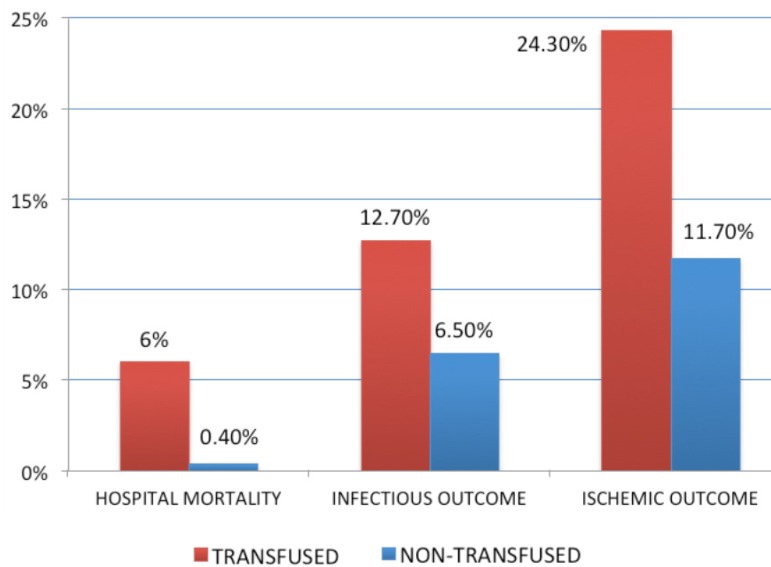
Outcomes in patients with EuroSCORE ≤ 2

When adjusted by a multivariate logistic model, the association between transfusion and the three co-primary outcomes was maintained in relation to composite infectious outcomes (OR 2.67, 95% CI 1.70 to 4.19; *P*<0.001), composite ischemic outcomes (OR 2.42, 95% CI 1.70 to 3.46; *P*<0.001) and hospital mortality (OR 3.07, 95% CI 1.53 to 6.13; *P*<0.001) (Figure 1).

In our study, other variables were identified as independent predictors of the three outcomes, as follows: mortality (creatinine clearance < 30%, age > 75 years, diabetes mellitus, family history of coronary artery disease and current smoking), infectious outcomes (creatinine clearance ≤ 30%, age ≥ 75 years, chronic obstructive pulmonary disease and high body mass index) and ischemic outcomes (family history of coronary artery disease and female gender).

Among the 17 surgeons who performed CABG during the study period, excluding those who performed fewer than 10 procedures (5 professionals), the transfusion rate ranged from 53% to 79% (*P*<0.001).

Table 3 shows data from the multivariate logistic analysis for the prediction of the three co-primary outcomes.

**Table 3:** Multivariate logistic analysis for the prediction of three co-primary outcomes.

	Mortality		Infectious outcome		Ischemic outcome	
	OR (95% CI)	*P*	OR (95% CI)	*P*	OR (95% CI)	*P*
Transfusion	3.07 (1.53-6.13)	0.002	2.67 (1.7-4.19)	< 0.001	2.42 (1.7-3.46)	< 0.001
Age						
65-75 years	1.38 (0.79-2.41)	0.257	1.36 (0.91-2.02)	0.136	1.22 (0.88-1.69)	0.231
≥ 75 years	3.37 (1.75-6.48)	< 0.001	2.24 (1.32-3.82)	0.003	1.43 (0.9-2.26)	0.126
Female	1.07 (0.64-1.79)	0.795	1.36 (0.92-2.01)	0.119	1.47 (1.07-2.02)	0.017
BMI, kg/m^2^	1.02 (0.97-1.07)	0.434	1.11 (1.07-1.15)	< 0.001	1.03 (0.99-1.06)	0.114
Preoperative Hb, g/dL	0.91 (0.78-1.06)	0.223	1.06 (0.94-1.19)	0.319	0.96 (0.87-1.05)	0.37
CrCl ≤ 30 mg/dL	2.98 (1.11-8.02)	0.030	2.99 (1.24-7.23)	0.015	0.42 (0.15-1.16)	0.93
SAH	2.32 (0.89-6.08)	0.086	1.46 (0.77-2.79)	0.245	1.54 (0.96-2.49)	0.076
Diabetes mellitus	1.73 (1.08-2.78)	0.023	1.25 (0.88-1.78)	0.206	1.29 (0.97-1.72)	0.08
Family history of CAD	2.36 (1.11-5)	0.025	0.91 (0.45-1.84)	0.797	1.81 (1.1-2.96)	0.019
Tobacco use						
Ex-smoker	1.17 (0.68-2.03)	0.566	0.99 (0.66-1.49)	0.975	0.85 (0.61-1.18)	0.328
Current smoker	2 (1.05-3.78)	0.034	1.5 (0.93-2.42)	0.096	1.08 (0.72-1.61)	0.712
COPD	1.33 (051-3.5)	0.558	2.17 (1.08-4.37)	0.03	1.49 (0.79-2.8)	0.221
Prior stroke	1.72 (0.71-4.19)	0.229	1.27 (0.61-2.63)	0.527	1.34 (0.73-2.48)	0.345
Prior TIA	1.14 (013-9.85)	0.906	0.53 (0.06-4.35)	0.555	0.29 (0.04-2.37)	0.247
Carotid disease ≥ 50%	0.54 (0.19-1.51)	0.242	1.53 (0.79-2.94)	0.206	1.3 (0.72-2.34)	0.38
Peripheral vascular disease	1.8 (0.9-3.61)	0.096	1.35 (0.76-2.4)	0.302	1.29 (0.79-2.11)	0.311
Prior AMI	1.29 (0.79-2.11)	0.311	1.2 (0.84-1.72)	0.324	1.07 (0.8-1.44)	0.643
Triarterial or trunk injury	1 (0.58-1.73)	0.996	1.26 (0.84-1.91)	0.267	1.21 (0.87-1.68)	0.267
EF < 50%	0.98 (0.6-1.62)	0.949	0.91 (0.63-1.32)	0.63	0.87 (0.64-1.18)	0.354

AMI=acute myocardial infarction; BMI=body mass index; CAD=coronary artery disease; CI 95%=95% confidence interval; COPD=chronic obstructive pulmonary disease; CrCl=creatinine clearance; EF=ejection fraction; Hb=hemoglobin; OR=odds ratio; SAH=systemic arterial hypertension; TIA=transitory ischemic attack

## DISCUSSION

This retrospective study of 1,378 consecutive patients undergoing CABG revealed an association between blood transfusion and composite infectious and ischemic outcomes and mortality. Transfused patients are, as a group, at higher surgical risk; however, when only low-risk patients were selected by EuroSCORE, mortality remained high in the transfused group.

A blood product transfusion rate of 63.9% was found in this study. When only patients receiving packed red blood cells, with or without other blood components, were considered, the proportion was 59.3%. This value is consistent with the findings of other studies^[9,10]^. However, a downward trend in transfusion rates and the number of transfused bags has been observed in recent years^[11]^.

We used a 7 g/dL as a threshold to transfusion, but, in case of critical clinical events, the transfusion was indicated at higher levels of hemoglobin.

There was great variability in transfusion frequency among different surgeons, although a significant portion of these transfusions was performed in the intensive care unit.

As expected, transfused patients were a group of patients with higher risk of death and comorbidities. However, even in the low-risk group, identified as a low EuroSCORE risk (below 2%), there was an association between transfusion and morbidity and mortality.

### Composite Infectious Outcomes

A composite infectious outcome, defined as sepsis, respiratory infection and surgical wound infection, was significantly more prevalent among patients undergoing blood transfusions (16.1% *vs.* 6%). This relationship has been demonstrated in several publications^[12]^ and is believed to be related to the socalled "immunomodulating effect" of transfusion. This includes changes in blood cell compositions, such as a decreased number of circulating lymphocytes, changes in the T *helper*/T *suppressor* lymphocyte ratio and immune cell activation^[13]^. These changes ultimately predispose the individual to susceptibility to infectious agents, particularly bacteria. Perioperative blood transfusion enhances the already established inflammatory status caused by cardiopulmonary bypass and surgical trauma, increasing the levels of circulating cytokines by up to 15 times^[14]^.

Some authors have suggested that the leukodepletion of blood products decreases cytokine levels, reducing the deleterious effects of transfusion, but this has not translated into improved clinical outcomes in subsequent studies^[15]^. Our study did not include this analysis due to its low frequency in our cohort and because it is not a common practice in Brazil.

### Composite Ischemic Outcomes

Following the findings of previous studies that have shown an association between transfusion and tissue ischemia in patients undergoing CABG, our results demonstrated a higher incidence of composite kidney failure, stroke and acute perioperative myocardial infarction among transfused patients (29.4% *vs.* 12.6%). How blood transfusions lead to ischemic complications in these patients is not fully understood, but it is believed that the previously described inflammatory changes are responsible for these findings. Additional molecular, biochemical and structural changes caused by the storage of bags lead to decreased concentrations of 2 3-diphosphoglycerate, fragility and lower red blood cell distensibility, reducing their capacity to supply oxygen to tissues^[16]^.

### Mortality

The risk of death was three times higher among transfused patients when compared to the non-transfused. The former significantly higher average EuroSCORE; older age; a higher prevalence of combined surgeries, severe coronary lesions, and kidney failure; and lower hemoglobin levels indicate that this is a group with higher preoperative morbidity. This observation could raise questions about the real effect of transfusion on outcomes. However, when adjusted for these and other variables, transfusion remains an independent risk factor for mortality and combined outcomes.

Other information contradicting the hypothesis that the higher mortality in the transfused group stems from patients' conditions being more severe was provided by an analysis of mortality in low-risk strata. When only patients with low logistic EuroSCORE (≤ 2%) were evaluated in both groups, the mortality rate among the transfused remained significantly higher.

### Low-Risk Group

The highest mortality rate, infection and ischemic outcomes in the transfusion group may not be due to transfusion, but due to complications after CABG in a high-risk group. This are important confounders in studies evaluating transfusion after CABG. Patients receiving blood products are part of a higher risk group, due to their high number of comorbidities. In addition, it is the situation that caused the major bleeding, with its tissue hypoxia complications, an additional surgical procedure to correct the bleeding and the possible adverse effects of transfusion. Transfused patients more frequently require inotropic and mechanical support. Despite careful statistical adjustments, it is possible that these factors caused additional risk.

When patients with low baseline risk profiles were selected by logistic EuroSCORE < 2%, confounding factors were reduced. In this scenario, significantly higher mortality remained in the group receiving transfusions, as well as higher ischemic and infectious outcomes, possibly due to tissue hypoxia complications, could be a possibility caused by anemia/hypoperfusion associated with the deleterious effects of transfusion.

A recent prospective, randomized study^[17]^ testing the use of liberal or restrictive transfusion strategies, defined by the use of a threshold for transfusion of 9.0 g/dL or 7.0 g/dL, did not show differences between the two subgroups. However, the difference in hemoglobin levels between the groups was only 1 g/dL.

As expected, the minimum postoperative hemoglobin level in transfused individuals was significantly lower than in patients who did not receive transfusions (7.9±1.2 *vs.* 8.9±1.3). These values, rather than a patient's actual clinical indication, guide transfusion indications in the operating room, especially in surgery involving cardiopulmonary bypass^[18]^. The use of a restrictive transfusion strategy has proven to be well tolerated by patients in various studies. In a randomized clinical trial of patients undergoing CABG, Bracey et al.^[19]^ reported that a reduction in the transfusion hemoglobin threshold to 8 mg/dL did not negatively affect outcomes and resulted in lower costs. In a large multicenter study of critically ill patients not undergoing cardiac surgery, Hebert et al.^[20]^ found that a restrictive strategy (hemoglobin < 7 mg/dL) could be superior to a liberal strategy (hemoglobin < 10 mg/ dL). Results such as these lend support to the global trend of the reduction of tolerated hemoglobin and hematocrit levels and hence the performance of fewer transfusions on these patients.

Although guidelines for transfusion practices in cardiac surgery have already been published^[21]^, and despite all the evidence made available during the last three decades of research, there remains great variability in the performance of transfusions among institutions and even among professionals in the same institution^[22]^. Over 20 years ago, Goodnough et al.^[23]^ study demonstrated significant variability in transfusion rates in 540 patients undergoing cardiac surgery in 18 institutions (17% to 100% for packed red blood cells). In 2010, Bennett-Guerrero et al.^[10]^ found similar rates (7.8% to 92.8%) in a retrospective analysis of CABGs performed in 2008 across 798 centers. This demonstrates the lack of consensus on the level of hematocrit required to ensure the benefits of transfusion over the potential risks. The indication for transfusion derives, above all, from the personal judgment of surgeons, anesthetists and clinicians, often to the detriment of an individual's clinical condition. It is the professionals, rather than the patients themselves, who do not tolerate low levels of hemoglobin and hematocrit.

### Study Limitations

The main limitation of the study relates to the reasons, aside from hemoglobin values, for blood transfusion. The reason for performing the transfusion, a surgical complication resulting in bleeding, may itself be associated with high morbidity and mortality, regardless of transfusion.

As this was a retrospective study, the results allowed us to evaluate the association between the transfusion variable and morbidity and mortality outcomes, but we cannot demonstrate causality among them. Another limitation, also related to the retrospective nature of this study, is the lack of data determining the period in which the transfusion and outcome occurred during hospitalization. Furthermore, this study used mortality from all causes as a primary outcome and did not differentiate between cardiac and non-cardiac causes of death. There is no data concerning transfusion of critical clinical patients, which are associated with higher incidence of complications.

## CONCLUSION

In this study, the transfusion of blood products was found to be harmful for most patients undergoing CABG, even in those with low baseline risk. Studies evaluating whether the higher incidence of complications in these patients is due to blood transfusion or to hypoxia complications are necessary to better understand transfusion practice.

**Table:** 

Authors' roles & responsibilities	
ICC	Substantial contributions to the conception or design of the work; or the acquisition, analysis, or interpretation of data for the work; final approval of the version to be published
VT	Substantial contributions to the conception or design of the work; or the acquisition, analysis, or interpretation of data for the work; final approval of the version to be published
HPM	Substantial contributions to the conception or design of the work; or the acquisition, analysis, or interpretation of data for the work; final approval of the version to be published
MCMC	Substantial contributions to the conception or design of the work; or the acquisition, analysis, or interpretation of data for the work; final approval of the version to be published
FAM	Substantial contributions to the conception or design of the work; or the acquisition, analysis, or interpretation of data for the work; final approval of the version to be published
GM	Substantial contributions to the conception or design of the work; or the acquisition, analysis, or interpretation of data for the work; final approval of the version to be published
JGTE	Substantial contributions to the conception or design of the work; or the acquisition, analysis, or interpretation of data for the work; final approval of the version to be published
HCCS	Substantial contributions to the conception or design of the work; or the acquisition, analysis, or interpretation of data for the work; final approval of the version to be published
CMCG	Substantial contributions to the conception or design of the work; or the acquisition, analysis, or interpretation of data for the work; final approval of the version to be published
PSF	Substantial contributions to the conception or design of the work; or the acquisition, analysis, or interpretation of data for the work; final approval of the version to be published
